# Ten years follow up of first degree relatives of type 1 diabetes patients: presence of autoimmune biomarkers and the progression to diabetes in a retrospective cohort

**DOI:** 10.20945/2359-3997000000370

**Published:** 2021-07-16

**Authors:** Isabella Sued Leão, Débora Batista Araujo, Bianca Barone, Joana Rodrigues Dantas, Matheus Victor de Souza Nolasco da Silva, Marina Oliveira Soares, Daniel Barretto Kendler, Rosane Kupfer, Lenita Zajdenverg, Melanie Rodacki

**Affiliations:** 1 Universidade Federal do Rio de Janeiro Departamento de Nutrologia Rio de Janeiro RJ Brasil Departamento de Nutrologia, Universidade Federal do Rio de Janeiro (UFRJ), Rio de Janeiro, RJ, Brasil; 2 Universidade Federal do Rio de Janeiro Faculdade de Medicina Rio de Janeiro RJ Brasil Faculdade de Medicina, Universidade Federal do Rio de Janeiro (UFRJ), Rio de Janeiro, RJ, Brasil; 3 Instituto Estadual de Diabetes e Endocrinologia Luiz Capriglione Rio de Janeiro RJ Brasil Instituto Estadual de Diabetes e Endocrinologia Luiz Capriglione (Iede), Rio de Janeiro, RJ, Brasil

**Keywords:** Autoimmunity, diabetes, ZnT8A, biomarker, type 1 diabetes

## Abstract

**Objective:**

The aim of the study was to assess the autoimmunity in first degrees relatives (FDR) of patients with type 1 diabetes (T1DM) and the progression to T1DM after 10 years of follow up in the Brazilian population.

**Subjects and methods:**

Non-diabetic FDR of T1DM patients were interviewed and blood was drawn for autoantibodies measurement (GADA, IA-2A, IAA, ZnT8A). Serum samples were analyzed by standard radioligand binding assays performed at the Federal University of Rio de Janeiro (GADA, IAA and IA2A), and at the Skäne University Hospital, Sweden (ZnT8A). The FDR were interviewed by phone after 10 years to determine if they had developed T1DM. Descriptive statistical analysis was performed and results were described as means and standard deviation (SD).

**Results:**

81 individuals were analyzed. Thirteen subjects had positive autoantibodies associated with T1DM.10 were positive for 1 autoantibody and 3 subjects were positive for multiple autoantibodies (1 of them showed positivity for 2 autoantibodies – GADA, ZnT8A – and the other two were positive for 3 autoantibodies – GADA, IA2A, ZnT8A). The 3 subjects with multiple positive autoantibodies developed T1DM within 10 years.

**Conclusions:**

In Brazilian FDR of T1DM patients, the positivity for multiple autoantibodies indicate a greater chance of progression to T1DM, similar to observed in Caucasians. ZnT8A was helpful in the risk assessment for T1DM development.

## INTRODUCTION

Type 1 diabetes mellitus (T1DM) is a chronic autoimmune disease caused by destruction of the insulin-producing pancreatic beta cells. This process leads to insulin deficiency and dysregulation of the glycemic metabolism, which causes long-term complications and increases the morbimortality of the disease.

The development of clinically apparent T1DM is usually preceded by the appearance of autoantibodies to islet autoantigens, such as glutamic acid decarboxylase (GADA),insulinoma-associated antigen 2/ICA512 (IA2A), insulin (IAA) and zinc transporter 8 (ZnT8A) ( 1 – 4 ). Several prospective studies have demonstrated that these antibodies usually appear years before the development of clinically apparent T1DM. Individuals who have developed two or more T1DM-related autoantibodies and are normoglycemic are classified as T1DM stage 1. As this disorder progresses, the patient will undergo T1DM stage 2 characterized by dysglycemia and two or more T1DM-associated autoantibodies. Finally, the individual will go through stage 3, which includes autoimmunity, dysglycemia and clinical symptoms and signs of diabetes ( 5 ).

The presence of autoimmunity does not necessarily indicate progression to T1DM stage 3. Positivity for a single autoantibody carries a relatively low risk of developing this condition, while the appearance of multiple autoantibodies (two or more) increases this risk to near certainty of progressing to diabetes (44% in 5 years, 77% in 10 years and almost 100% during the whole lifetime) ( 4 – 14 ). Positivity for multiple autoantibodies is used as a biomarker in risk scores for the development of T1DM, in prevention and intervention studies, along with HLA haplotypes, the first-phase insulin response, and impaired glucose tolerance ( 15 ). However, most studies in this field included only Caucasians and it is important to investigate whether the behavior of these humoral markers of diabetes follows the same pattern in multiethnic populations, when compared with Caucasians. The Brazilian population is ethnically diverse and considered one of the most heterogeneous in the world as significant admixtures within each ethnic group have been reported.

The aim of this study was to assess the autoimmunity in first degree relatives (FDRs) of patients with T1DM and the progression to T1DM after 10 years in the multiethnic Brazilian population. We hypothesized that, despite the multiethnic background of the Brazilian population, the study sample would behave like Caucasians.

## SUBJECTS AND METHODS

In this retrospective cohort, subjects were recruited from May 2006 to May 2009. The sample size was defined by convenience. Data were collected in the same period. Ten years after enrollment (between 2016 and 2019), a follow-up contact by telephone was made to assess the outcome, which was the diagnosis of T1DM. Data analysis was carried out in 2019.

### Subjects

Brazilian non-diabetic FDRs of patients with T1DM who were being followed up at the Diabetes Outpatient Clinic of the Federal University of Rio de Janeiro (UFRJ) and the State Institute of Diabetes and Endocrinology Luiz Capriglione (IEDE) were invited to participate in this study. The recruitment period and data collection were from May 2006 to May 2009. The initial invitation to participate in the study was made through telephone contact or at the end of the routine consultations of patients being monitored in the units mentioned above. FDRs of patients with T1DM were included.

The inclusion criteria for selection of the participants were being a child or sibling of a patient with T1DM and aged between 5 and 40 years at baseline. Only one FDR per patient was included. Exclusion criteria were confirmed diabetes mellitus or, in the case of siblings, having only one parent in common.

As for ethnicity, we chose to divide our population into Caucasians and non-Caucasians (mostly Afro-descendants), because there is a greater genetic similarity between Afro-Brazilians and Indians than with Euro-Brazilians, according to a study by Palatnik and cols. ( 16 ) This division was based on the FDR phenotype and family background.

The project was approved by the institutional ethical committee. All participants signed an informed consent form. For underage participants, the consent form was signed by a guardian.

### Clinical and laboratory evaluation

Participants were interviewed and blood was drawn for autoantibodies measurement (GADA, IA-2A, IAA and ZnT8A). Serum samples were analyzed by a standard radioligand binding assay (RBA) for GAD, insulin and tyrosine phosphatase A (GADA, IAA and IA2A, respectively), which were performed at the Federal University of Rio de Janeiro, Rio de Janeiro, Brazil. The RBA to analyze the samples for the Zn-T8A comprised the three individual Zn-T8A variants (ZnT8RA, ZnT8WA and ZnT8QA) as well as the ZnT8TripleA assay, which were both developed at the Department of Clinical Sciences, Skäne University Hospital, Malmö, Sweden. Cut-off values for the positive test were set to 1.0 U/mL for GADA, IAA and IA2A, 75 U/mL for ZnT8RA and ZnT8WA, 100 U/mL for ZnT8QA and 60 U/mL for the ZnT8ATriple assay (ZnT8AQRW).

Ten years after the autoantibodies measurement, the FDRs were interviewed by phone to determine if they had developed T1DM or not. The outcome was the development of T1DM during the 10 year follow-up period. American Diabetes Association criteria were used to define diabetes. The presence of hallmark symptoms, such as polyuria, polydipsia and polyphagia and/or of weight loss before the diagnosis, ketoacidosis at diagnosis, abrupt development of diabetes and/or the need for insulin therapy were used as criteria to define T1DM, associated with the presence of autoantibodies ( 17 ).

### Statistical analysis

Descriptive statistical analysis was performed and results are presented as means and standard deviation (SD).

## RESULTS

### Study population

A total of 81 FDRs of patients with T1DM were analyzed, (50 siblings and 31 offspring). The median age of the study group was 20 years old SD (range:5 to 46 years old). Most subjects were non-Caucasians (59.2%) and females (58%).

The population of T1DM patients also had a total of 81 patients. The mean age was 30.19 ± 11.23 years, with a predominance of non-Caucasians (55.7%) and women (61.5%). The mean age at T1DM diagnosis was 18.63 ± 11.31.

### Autoantibodies measurement

Thirteen subjects (16%) had autoantibodies associated with T1DM (GADA, IAA, IA2 and/or anti-ZnT8). Ten subjects (76.9% of those) were positive for only one autoantibody, with predominance of GADA as a single autoantibody. (GADA 70%, IA2A 20%, IAA 10% in these individuals). The remaining 3 subjects (23.1% of those with positive antibodies) were positive for multiple autoantibodies.

### Ten-year follow-up

Among 81 relatives screened between 2006 and 2009, 16 were lost to follow-up (19%), including 1 subject positive for a single autoantibody (IAA). Twelve of the 65 remaining FDRs (18.4%) had positive autoantibodies. The characteristics of the FDRs with positive autoantibodies are shown in Table 1 .

**Table 1:** Characteristics of the FDR with positive autoantibodies

FDR	Age at autoantibody assessment	Sex	Ethnicity	Relationship	Autoantibodies	Diabetes	Age at T1DM diagnosis
HOS 1	12	Male	Non-Caucasian	Offspring (father DM)	GADA (39.73 UI/mL)	No	N/A
MSS 2	22	Male	Non-Caucasian	Offspring (mother DM)	GADA (45.31 UI/mL)	No	N/A
BLR 3	08	Female	Non-Caucasian	Offspring (father DM)	GADA (1.34 UI/mL)	No	N/A
MVP 4	33	Male	Non-Caucasian	Sibling	GADA (2.02 UI/mL)	Pre-diabetes	N/A
MSC 5	08	Male	Non-Caucasian	Sibling	IAA (1.17 UI/mL)	Lost follow up	N/A
RNS 6	28	Female	Caucasian	Sibling	GADA (1.16 UI/mL)	No	N/A
FSP 7	18	Male	Non-Caucasian	Sibling	GADA (1.4 UI/mL) IA2A (12.0 UI/mL) ZnT8AQRW (3,703 UI/mL) ZnT8AQ (2,229 UI/mL) ZnT8AR (2,810 UI/mL) ZnT8AW (2,608 UI/mL)	Yes	21
RBGF 8	20	Female	Caucasian	Sibling	IA2A (1.4 UI/mL)	No	N/A
MCMS 9	37	Female	Caucasian	Sibling	IA2A (1.3 UI/mL)	No	N/A
FSS 10	09	Female	Caucasian	Offspring (father DM)	GADA (56.73 UI/mL)	No	N/A
RTP 11	29	Male	Caucasian	Sibling	GADA (61.38 UI/mL) ZnT8AQRW (772 UI/mL) ZnT8AR (3368 UI/mL)	Yes	35
FPL 12	18	Male	Caucasian	Sibling	GADA (2.31 UI/mL)	No	N/A
JRN 13	18	Female	Caucasian	Sibling	GADA (28.02 UI/mL) IA2A (8.77 UI/mL) ZnT8AQRW (197 UI/mL) ZnT8AQ (243 UI/mL) ZnT8AR (262 UI/mL) ZnT8AW (157 UI/mL)	Yes	19

Reference values: GADA < 1.0 U/mL; IAA < 1.0 U/mL; IA2A < 1.0 U/mL; ZnT8RA < 75 U/mL; ZnT8WA < 75 U/mL; ZnT8QA < 100 U/mL; ZnT8ATriple assay (ZnT8AQRW) < 60 U/mL.

FDR: first degree relatives; N/A: not apply.

Among the 65 FDRs who underwent follow-up, GADA was the most prevalent autoantibody, present in 9 cases. Four FDRs were positive for IA2A, 3 for ZnT8A and 1 for IAA. Of these 12 positive FDR, 1 had 2 positive autoantibodies (GADA and ZnT8A) and 2 had 3 positive autoantibodies (GADA, IA2A and ZnT8A). Three patients were negative for GADA or IAA, but were not tested for IA2A or ZnT8A. The FDR lost to follow-up with positivity for IAA tested negative for the other autoantibodies.

Three subjects with positive autoantibodies developed T1DM within 10 years. The T1DM diagnoses were established after 1, 3 and 6 years of the autoantibodies measurements. Their median age was 21.66 years old ± 5.18 at baseline and all were siblings of patients with T1DM. All these subjects had more than one positive autoantibody (2 tested positive for GADA, IA2A and ZnT8A and 1 for GADA and ZnT8A). The only individuals who developed T1DM during follow-up were those who had multiple autoantibodies (2 or more). Additionally, one subject with GADA had pre-diabetes mellitus, and had gained 10 kg in the past 10 years (BMI 38.10 kg/m^2^). In this case, low GADA titers were observed (2.02 U/mL).

**Figure 1:**
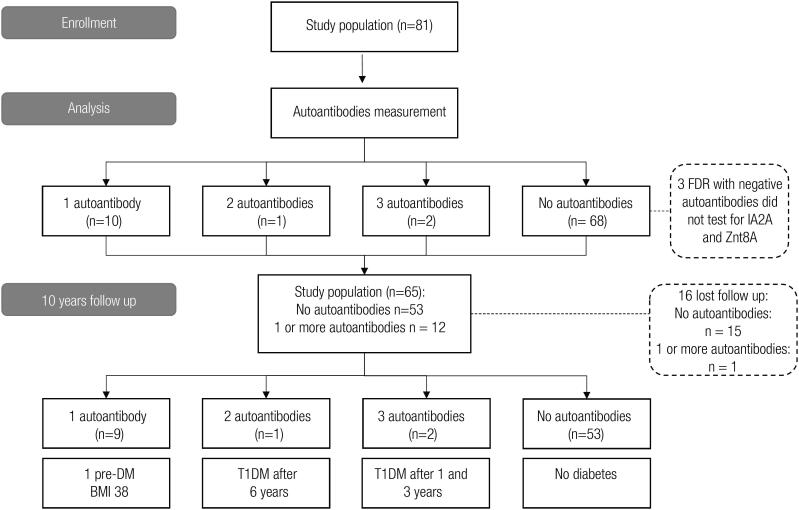
Flow diagram with the stages of the study – FDR: first degree relatives; DM: diabetes mellitus; T1DM: Type 1 diabetes mellitus.

The results of the study are summarized in Figure 1 .

## DISCUSSION

In the present study, we evaluated the risk of developing T1DM in FDRs of T1DM patients based on the detection of positive autoantibodies. Our work assessed a wide age range and a multiethnic population, to identify if the findings for the Caucasians were also valid for the Brazilian population.

Of the 81 initial FDRs, 16% had one or more autoantibodies. The incidence of positive autoantibodies in our population was higher than that found in most populations studied worldwide. ( 7 , 11 , 14 , 18 ) and also differs from another Brazilian study ( 19 ). This may be a unique characteristic of our population, because the FDR selection criteria were similar to those of other studies in the literature ( 18 ). Among FDRs with positive autoantibodies, 75% were positive for only one autoantibody and did not develop T1DM within 10 years. Most of these individuals showed the autoantibody at low titers. The predominance of positivity for a single autoantibody is not unique to the Brazilian population and has also been found in other populations, such as in the DPT-1, BABYDIAB, TEDDY and TrialNET study groups ( 6 , 7 , 11 , 14 ). The only subjects who progressed to T1DM in this period were those with two or more positive autoantibodies. This finding is in agreement with other studies performed mostly with Caucasians, which showed that the presence of a single autoantibody does not represent a high risk for the development of T1DM. On the other hand, the presence of multiple autoantibodies appears to be the hallmark of a “point of no return” in the T1DM pathogenic process, which signals the beginning of the preclinical stage of the disease ( 4 , 14 , 20 ).

ZnT8A is considered an additional diagnostic marker of T1DM that improves the overall autoantibody sensitivity. It reduced the proportion of patients with negative autoantibodies and increased the diagnostic sensitivity to over 90% for new onset cases of T1DM in Caucasians and in a Brazilian population study and to 80% in India ( 21 – 24 ). Furthermore, there is an independent relationship between the risk of developing T1DM and ZnT8A that favors its use in screening, especially in high-risk populations, such as FDRs of T1DM patients ( 23 , 25 – 27 ). Because most FDRs who undergo GADA, IAA and IA2A combined measurement are positive for only one of these autoantibodies, the addition of a ZnT8A test to the screening process helps to identify individuals with another positive antibody, which would represent a higher risk for T1DM ( 27 ). Interestingly, among the three subjects that developed T1DM positivity for more than one autoantibody, one would not have been detected if ZnT8A measurements had not been performed. Therefore, the ZnT8A positivity increased the number of individuals with positivity for more than one autoantibody.

Lastly, the positivity for ZnT8A conferred in the literature a greater risk of rapid progression to T1DM ( 15 ). The DAISY study showed a significantly greater risk of developing T1DM in 5 years in individuals who positive for ZnT8A when compared with those who did not exhibit positivity ( 8 , 21 ). In addition, the association of ZnT8A with IA2A proved to be the most sensitive combination for detecting FDRs with a high risk for rapid progression to T1DM ( 28 , 29 ). In this study, a fast evolution to clinical T1DM (1 and 3 years) occurred in those with ZnT8A and IA2A.

This study has some limitations. First, the sample size was small. Despite this, our results were consistent with those of other studies in the literature and the selection criteria were broad, similar to those of the DAISY ( 18 ) and TEDDY studies ( 11 ). Nevertheless, our study's population showed a higher prevalence of autoantibodies compared with other populations. This can be explained by differences in the population assessed in other studies. BABYDIAB ( 7 ) evaluated only offspring and TrialNET ( 14 ) also evaluated second and third degree relatives of T1DM patients, which can reduce the prevalence of autoantibodies in the selected population. Alves et al did not evaluate ZnT8A in a previous study with the Brazilian population ( 19 ), which may also have reduced the number of FDRs with positive autoantibodies. Moreover, our study lacked the periodic repetition of antibodies measurement and pancreatic function studies. This could be the reason for the higher prevalence of autoantibodies in this study population. Of the FDRs, 70% had low titers of a single autoantibody that might not be persistent and could have disappeared in a second evaluation. However, this publication brings important information to the field, as there is a lack of data on autoimmunity in T1DM in multiethnic populations such as the Brazilian population. A study with a larger number of participants and periodic repetition of antibodies measurement and pancreatic function studies is still required to corroborate our findings and evaluate the external validity of these results. There was a loss to follow-up in 19% patients within 10 years, including one positive for IAA. This loss to follow-up is within the expected average for long studies. One more limitation was the evaluation of progression to T1DM, which was performed only by telephone contact and not according to serum glycemia. This might resulted in underestimating the incidence of diabetes. However, diagnosis of T1DM through occasional glucose measurement is unlikely and patients were asked about specific details of T1DM at the time of diagnosis and insulin treatment, which makes the diagnosis of T1DM more reliable. Moreover three individuals were not tested for IAA and ZnT8A, which precluded us from concluding whether they were positive for multiple autoantibodies or not. Finally, it was not possible to assess the risk of developing T1DM by DPTRS or by the Genetic Risk Score due to lack of BMI and C peptide values and lack of specific genetic data ( 15 , 30 ).

In conclusion, this study demonstrated that positivity for only one autoantibody does not seem to confer a greater risk for developing T1DM in the Brazilian multiethnic population, whereas two or more markers indicate a greater chance of progression to the disease, which is similar to what was observed in Caucasians. ZnT8A was helpful for stratification in our population, because it was present in all three cases with multiple antibodies and its measurement allowed for the detection of multiple antibodies in one of them. Further studies are necessary to understand the role of ZnT8A in larger studies and other populations.
